# Intravaginal eletrical stimulation for bladder training method

**DOI:** 10.1590/S1677-5538.IBJU.2021.0161.1

**Published:** 2021-10-01

**Authors:** Cássio L. Z. Riccetto

**Affiliations:** 1 Universidade Estadual de Campinas Faculdade de Ciências Médicas Divisão de Urologia Feminina CampinasSP Brasil Divisão de Urologia Feminina - Faculdade de Ciências Médicas da Universidade Estadual de Campinas – UNICAMP, Campinas, SP, Brasil

## COMMENT

The pathophysiological mechanisms involved in the symptoms of overactive bladder syndrome are varied. Thus, despite the current guidelines are organized in steps for treatment (1), based on the complexity of the clinical presentation, it is fair to consider a multimodal and individualized approach adjusted to the particular clinical aspects of each patient since the since the beginning of the treatment (2).

The use of electrical stimulation in the treatment of overactive bladder has been proposed for several years (3), and has been described in various modalities, such as: (a) intravaginal stimulation though a vaginal probe; (b) presacral stimulation through surface electrodes; (c) electrical stimulation of the tibial nerve and; (d) sacral neuromodulation, which is currently the best studied therapy based on electrical stimulation, with good results in more complex cases (4) and even for selected neurogenic patients (5). Limitations against the first three are: the intermittent pattern of the treatment; dependence on going several times to a specialized center for treatment; and the lack of studies on long-term outcomes. On the other hand, they are relatively low-cost methods that allow the physiotherapist to be added to the treatment in a more significant way.

Although proposed for several years, the effectiveness of bladder training on symptoms of overactive bladder is still poorly studied in the literature (6) as the authors described in their introduction. Most studies on bladder training associate its use to a pelvic floor muscle training program, which seems logical, but making it difficult to assess bladder training effectiveness as an isolated treatment (7). Furthermore, the lack of standardization of bladder training strategies also makes it difficult to compare the results presented in the studies.

In the present prospective randomized trial (8), the authors described their bladder training method, which was based on three stages: firstly patient understanding of the role of the pelvic floor in female urinary continence; then, she was invited to learn how to suppress of urgency through contraction of the pelvic floor, associated with techniques of respiratory rhythm control, concentration and self-motivation; and further a programmed urination protocol was applied, with increasing micturition's intervals. The authors concluded that the association of bladder training with intravaginal electrical stimulation was superior to isolated electrical stimulation in the treatment of overactive bladder. This contribution is relevant, as this condition has been becoming higlyly prevalent in women. Moreover, it serves as a guide to the multidisciplinary team, in order to not ignore any step in overactive bladder approach.
